# Modeling the Relationship between Fluorodeoxyglucose Uptake and Tumor Radioresistance as a Function of the Tumor Microenvironment

**DOI:** 10.1155/2014/847162

**Published:** 2014-09-08

**Authors:** Jeho Jeong, Joseph O. Deasy

**Affiliations:** The Department of Medical Physics, Memorial Sloan Kettering Cancer Center, New York, NY 10065, USA

## Abstract

High fluorodeoxyglucose positron emission tomography (FDG-PET) uptake in tumors has often been correlated with increasing local failure and shorter overall survival, but the radiobiological mechanisms of this uptake are unclear. We explore the relationship between FDG-PET uptake and tumor radioresistance using a mechanistic model that considers cellular status as a function of microenvironmental conditions, including proliferating cells with access to oxygen and glucose, metabolically active cells with access to glucose but not oxygen, and severely hypoxic cells that are starving. However, it is unclear what the precise uptake levels of glucose should be for cells that receive oxygen and glucose versus cells that only receive glucose. Different potential FDG uptake profiles, as a function of the microenvironment, were simulated. Predicted tumor doses for 50% control (TD_50_) in 2 Gy fractions were estimated for each assumed uptake profile and for various possible cell mixtures. The results support the hypothesis of an increased avidity of FDG for cells in the intermediate stress state (those receiving glucose but not oxygen) compared to well-oxygenated (and proliferating) cells.

## 1. Introduction

As the most commonly used functional imaging modality in oncology, ^18^F-labeled fluorodeoxyglucose positron emission tomography (FDG-PET) has played a valuable role since first being synthesized in 1978 [1]. Most malignant tumors exhibit an elevated glucose uptake and FDG-PET, as a close glucose chemical analogy, results in an image of glucose uptake in the patient, offering unique information for cancer detection, staging, target definition, and response monitoring [2, 3].

As a significant predictor of prognosis in radiation therapy (RT), FDG uptake seems to reflect an increased radioresistance; however, this is poorly understood. Many clinical outcome analyses have verified that high uptake of FDG in a tumor is correlated with increased local failure and shorter survival for many tumor sites, as summarized in several meta-analyses [4–7]. Therefore, FDG-avid regions in a tumor are recognized as a possible target for dose escalation to compensate for the radioresistance [8, 9]. Recently, utilizing a novel meta-analysis tool, we showed that FDG-avid head and neck tumors require about 20% more doses to equalize the local control rate with FDG nonavid tumors [10], although tumor size confounded that analysis to an unknown extent.

Enhanced glycolysis of tumor cells is certainly related to hypoxia, because hypoxic cells produce energy (in the form of ATP molecules) through glycolysis, without oxygen. However, it is also known that tumor cells can show increased glycolysis even in the presence of oxygen, compared to normal cells (the “Warberg effect”) [11, 12]. The oncolytic appetite for glycolysis is thought to be caused by a number of genetic or possibly epigenetic changes that drive malignancy [13, 14].

Many studies have been carried out to correlate FDG uptake with various physiological parameters, such as hypoxia, proliferation, blood flow, histology, and differentiation, utilizing FDG-PET and immunohistochemical methods [15–20]. However, although several studies have shown the relationship between the FDG uptake and hypoxia or proliferation, the underlying mechanism of FDG uptake in a tumor is still unclear.

In this study, we do not try to resolve the detailed mechanism of FDG uptake. However, we do test various assumptions correlating FDG uptake (and presumably glucose consumption) with local cell microenvironmental conditions. The model is an attempt to incorporate known radiobiological effects that have been established as being important to understand radiotherapy treatment response, such as varying access to oxygen and glucose as well as the basic mathematical features of tumors, including variable growth fractions and cell loss factors. The key starting point of the model is that there is a limited amount of chemical resources for each tumor subvolume and that this level of resources is assumed to remain constant over a course of radiotherapy. We therefore used the model to determine the assumptions relating FDG uptake to the underlying cell compartments that best fits the observed correspondence between FDG uptake and reduced local control.

## 2. Methods

### 2.1. State-Driven Tumor Response Model

**Figure 1:**
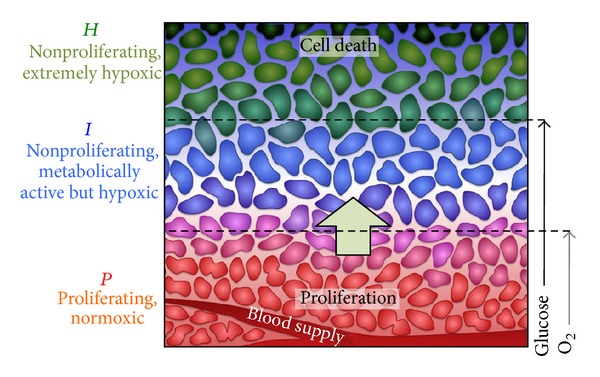
Cartoon of the microenvironment with respect to the blood supply. Due to a limited blood supply, there are limited supplies of the oxygen and the key nutrient, glucose. Cells with adequate oxygen and glucose are actively proliferating, while cells distant from vessels are starving and dying. In the model, these populations of cells were simplified into three different compartments (*P*,* I*, and* H*) that have different levels of proliferation, hypoxia, cell death, and radiosensitivity. Note that uniform levels of glucose and O_2_ were assumed for each compartment.

To explore the potential relationship between FDG-PET uptake and classical radiobiological mechanisms, a previously developed state-based tumor response model was used [21]. In the mechanistic model, a tumor was assumed to be comprised of many small tumorlets of a PET-voxel-comparable size (4 × 4 × 4 mm^3^). Each tumorlet is comprised of three subpopulations of cells based on the level of proliferation, hypoxia, and cell loss, which is thought to be related to the available amount of oxygen and glucose, as shown by Kiran et al. [22].  Figure 1 shows the three compartments of a tumorlet in a typical tumor microenvironment. Proliferation was assumed to take place only in the* P*-compartment, wherein cells have access to glucose and oxygen, whereas cell loss in the absence of treatment only takes place in the* H*-compartment, wherein cells have no access to either oxygen or glucose. The intermediate cells in the* I*-compartment, which have access to glucose but not oxygen, were assumed to be metabolically active but do not proliferate.

After RT begins, damaged cells (as deterministically calculated by linear-quadratic model) become doomed according to a compartment-specific radiosensitivity, leading to mitotic cell death when doomed cells are attempting to proliferate in the* P*-compartment. As doomed cells die, metabolically active but hypoxic cells in the* I*-compartment receive oxygen and move into the* P*-compartment, causing reoxygenation. The model assumes a locally-constant blood supply over the course of RT. The transition of cells between compartments is determined dynamically, with the* P*-compartment always taking as many cells from the* I*-compartment as can be supported.

As a discrete-time simulation algorithm, the model keeps updating the number of cells in each compartment in a small time step. In each time step, a fraction of cells in the* P*-compartment proliferate, cell loss takes place in the* H*-compartment, the doomed cells in the* P*-compartment die probabilistically following mitosis, and the cells are recompartmentalized based on the capacity of each compartment. At the time of RT fraction, a fraction of viable cells in each compartment becomes doomed according to compartment-specific radiosensitivity value.

The model can be used to evaluate clinically important phenomena, including fraction size effects, the reoxygenation effect, repopulation effects, and tumor regression. Details of the mathematical model are available elsewhere [21].

### 2.2. Hypothetical FDG Uptake Patterns

**Figure 2:**
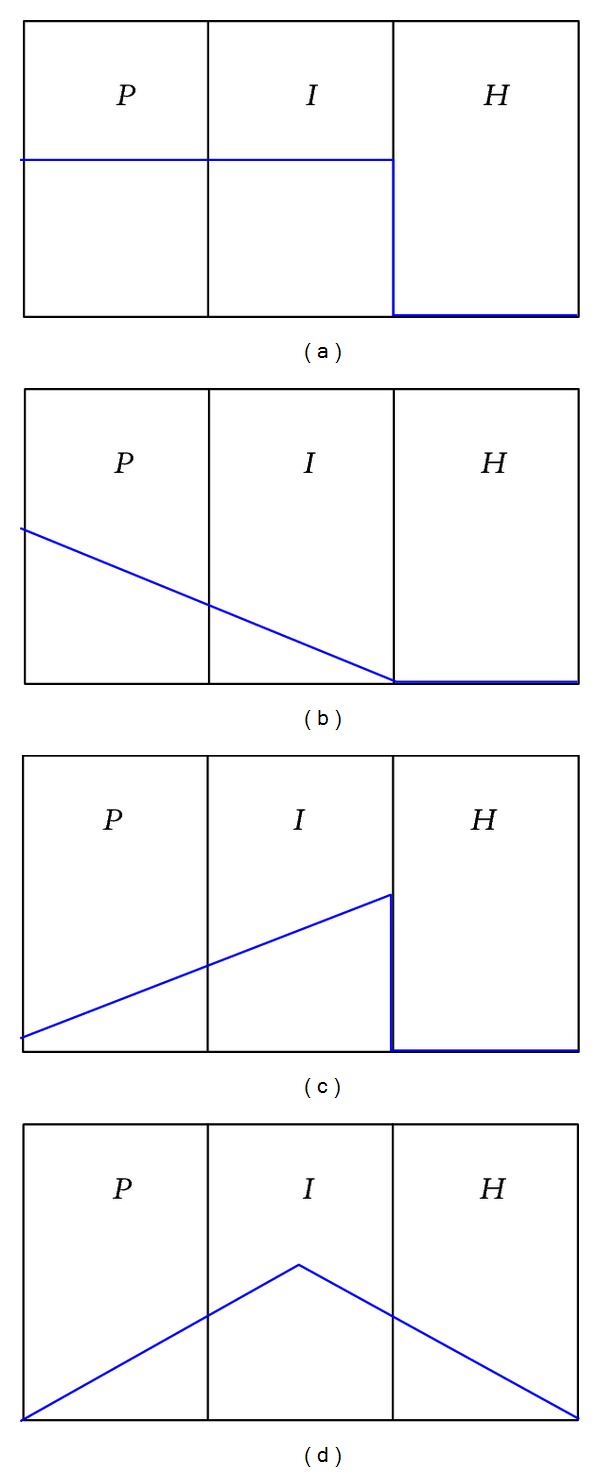
Assumed FDG uptake patterns in each compartment. We use straight lines to emphasize that uptake probably varies across compartments, even though this variation is simplified in the model. The FDG uptake patterns were assumed to be (a) proportional to the total number of viable cells (*pattern I*), (b) associated mainly with proliferation with a minor contribution from intermediate hypoxic cells (*pattern II*), (c) associated mainly with intermediate hypoxia with a minor contribution from proliferating cells (*pattern III*), and (d) associated mainly with intermediate hypoxic cells with minor contributions from proliferating and extremely hypoxic/dying cells (*pattern IV*). Note that the sizes of the three compartments are variable depending on the microenvironmental conditions, although they are shown with the same size in the figure.

We consider different relative uptakes of FDG within the three compartments. The subpopulations of tumor cells in the model were distinguished based on oxygen and glucose availability [22]: only the* P*- and* I*-compartments were thought to be associated with FDG uptake given the model assumptions. Based on correlation studies between FDG and physiological factors, three different potential relationships between FDG uptake and cell subpopulations are considered here: (1) FDG uptake is proportional to the total number of metabolically viable cells (*pattern I*); (2) FDG uptake is associated mainly with proliferating cells with a reduced contribution from intermediate cells (*pattern II*); and (3) the FDG uptake is associated mainly with the intermediate cells with a reduced contribution from proliferating cells (*pattern III*), all shown in Figure 2. A reduced contribution from extremely hypoxic (starving and dying) cells in the* H*-compartment was additionally tested (*uptake pattern IV*).

### 2.3. Model Simulation

The model simulations were performed for a tumorlet treated with a standard RT regime of 2 Gy/fx (5 fx/week). The size of tumorlet was set to be 64 mm^3^ (based on typical PET-voxel size) with 6.4 × 10^7^ cells, assuming the tumor cell density of 10^6^ mm^−3^. Only a small subset of cells is known to have stem-cell-like property and 1% of viable cells were assumed to be clonogenic cells in the model [23]. Based on previous work [21], relevant parameter values for head and neck squamous cell carcinoma (HNSCC) were used, including the radiosensitivity of the* P*-compartment (*α*_*p*_ = 0.382 Gy^−1^, *α*/*β* = 6.63 Gy). Hypoxic cells in the* I*- and* H*-compartments are considered to be only in the G0/G1-phase and the OER values for the* I*- and* H*-compartments were assumed to be 2 and 1.37, respectively, considering the lower OER of the G0/G1-phase and reduced repair capability of chronically hypoxic/highly stressed cells in the* H*-compartment [24].

The initial distribution of cells in each compartment is determined based on the presumed growth fraction (GF) and cell loss factor (CLF). As the GF increases, more cells are in proliferation and the* P*-compartment becomes larger. Given a higher CLF, more cells are starving and dying, resulting in a larger* H*-compartment. To consider a wide range of possible microenvironmental conditions, all possible ranges of initial cell distributions were simulated, based on various combinations of GFs and CLFs (1315 combinations). The parameter values for different compartments and the ranges of GF and CLF used in the simulation are summarized in Table 1.

### 2.4. Correlation between FDG Uptake and TD_50_

For each assumed FDG uptake pattern, the FDG uptake values were quantified for all possible ranges of initial distributions of cells in each compartment. Then, simulations were performed to determine the tumor dose for 50% control (TD_50_) for all the possible ranges of initial cell distributions. The TD_50_ value was estimated from the Poisson formalism based on the total number of clonogenic cells in the tumorlet. Finally, we correlated the resulting FDG uptake values with the corresponding TD_50_ values and determined the linear correlation coefficient for comparison purposes.

## 3. Results

**Figure 3:**
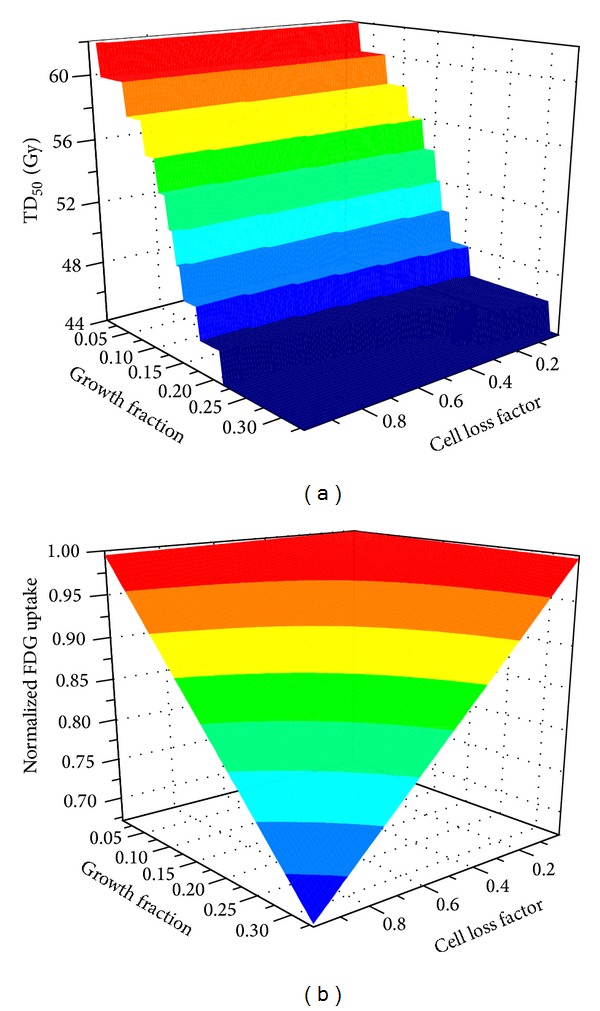
(a) Model predicted tumor dose for 50% control (TD_50_) assuming 2 Gy/fx and (b) normalized FDG uptake (for* pattern I*) for all possible initial conditions as a function of growth fraction (GF) and cell loss factor (CLF).

Estimated TD_50_ values for all possible ranges of initial conditions are shown in Figure 3(a). TD_50_ increased as the growth fraction decreased, due to a larger fraction of cells in the hypoxic compartments. TD_50_ decreased as the cell loss factor increased but the dependency of TD_50_ on the cell loss factor was much less significant compared to dependence on the growth fraction. The FDG uptake value was also quantified for all possible initial conditions based on the hypothesized FDG uptake patterns, as shown in Figure 3(b) for the uptake* pattern I*.

**Figure 4:**
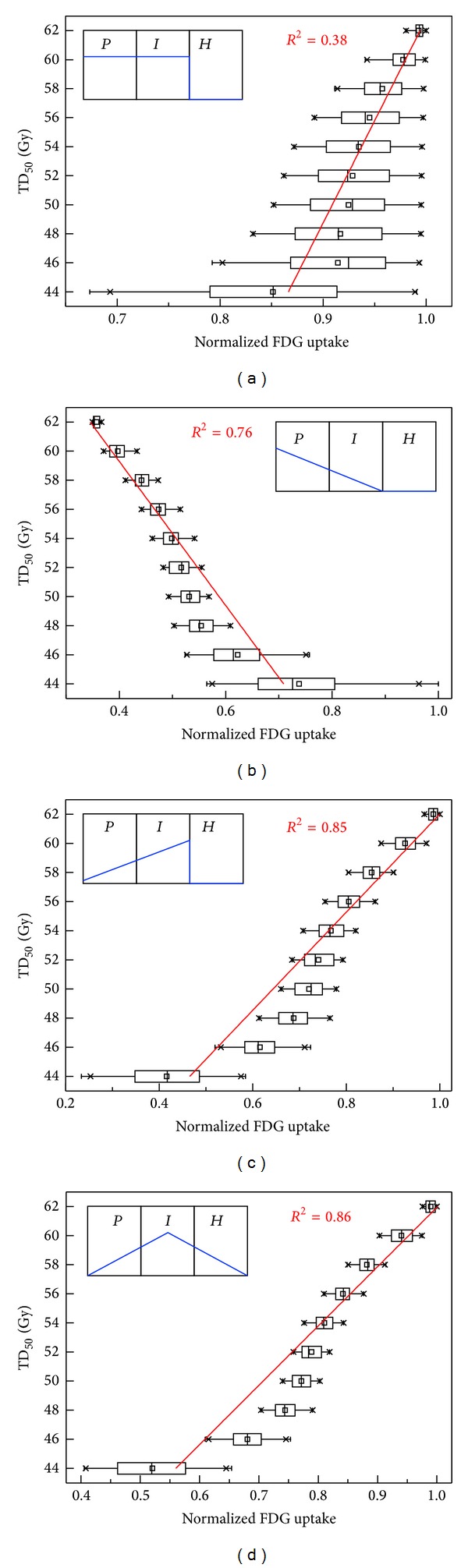
Correlations between model predicted tumor dose for 50% control (TD_50_) in 2 Gy/fx and FDG uptake (normalized to maximum) for four different hypothetical uptake patterns. For each plot, all possible ranges of initial distributions of cells were simulated based on various combinations of growth fractions and cell loss factors. Note that each box in the plot represents 25th to 75th percentile of the dataset with tails for whole range.

Hypothetical correlations between FDG uptake levels and corresponding TD_50_ values are shown in Figure 4, for the four different hypothetical FDG uptake patterns. For the first assumed pattern (*pattern I*), where the FDG uptake was assumed to be proportional to the total number of viable cells, only a weak positive correlation was observed between FDG uptake and TD_50_ value with a coefficient of determination (*R* ^2^) of 0.38. When the uptake pattern was assumed to be mainly associated with cell proliferation with a minor contribution from intermediate cells (*pattern II*), a significant negative correlation existed between FDG uptake and TD_50_ (*R* ^2^ = 0.76). For the hypothesis that metabolically-viable hypoxic cells in the* I*-compartment are avid for FDG uptake (*pattern III*), a strong positive correlation resulted (*R* ^2^ = 0.85). Inclusion of a reduced contribution from extremely hypoxic/starving cells in the* H*-compartment (*pattern IV*) yielded almost the same result as uptake* pattern III*, but with a slightly higher value of coefficient of determination (*R* ^2^ = 0.86), because the cells in* H*-compartment are either dying or reoxygenated into the* I*-compartment during the course of RT.

**Figure 5:**
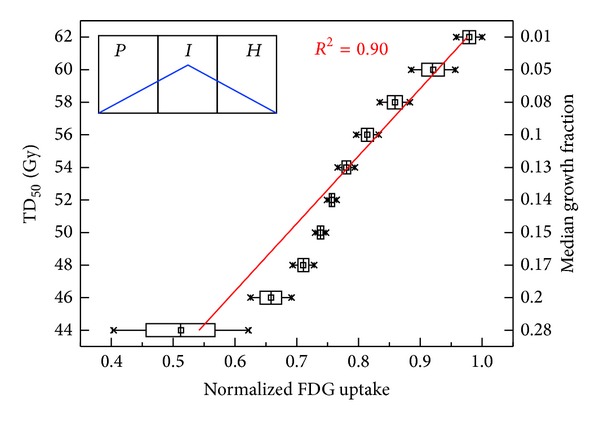
Model predicted tumor dose for 50% control (TD_50_) in 2 Gy/fx versus FDG uptake (normalized to maximum) with median growth fraction value for each TD_50_ group (right axis) for FDG uptake* pattern IV* with fixed cell loss factor of 0.9. Note that each box in the plot represents 25th to 75th percentile of the dataset with tails for whole range.

To better understand the potential relationship between FDG uptake and growth fraction (GF), the correlation between FDG uptake (for* pattern IV*) and TD_50_ was evaluated for a fixed cell loss factor of 0.9, as shown in Figure 5. For the assumed uptake pattern, the FDG uptake was inversely correlated with the median GF in each bin.

## 4. Discussion

Potential causes for the observed clinical correlation between FDG and radioresistance was explored using a mathematical model, in which classical radiobiological mechanisms were incorporated. Several different FDG uptake patterns were explored. Among the assumed FDG uptake patterns, when the metabolically viable hypoxic compartment (the* I*-compartment) was assumed to dominate the FDG uptake (uptake* pattern III *or* IV*), a significant positive correlation between FDG uptake and the required TD_50_ was observed (*R* ^2^ = 0.85 or 0.86, resp.), implying that the increased cellular radioresistance due to chronic hypoxia may be the cause of the clinically observed increase in tumor radioresistance.

**Figure 6:**
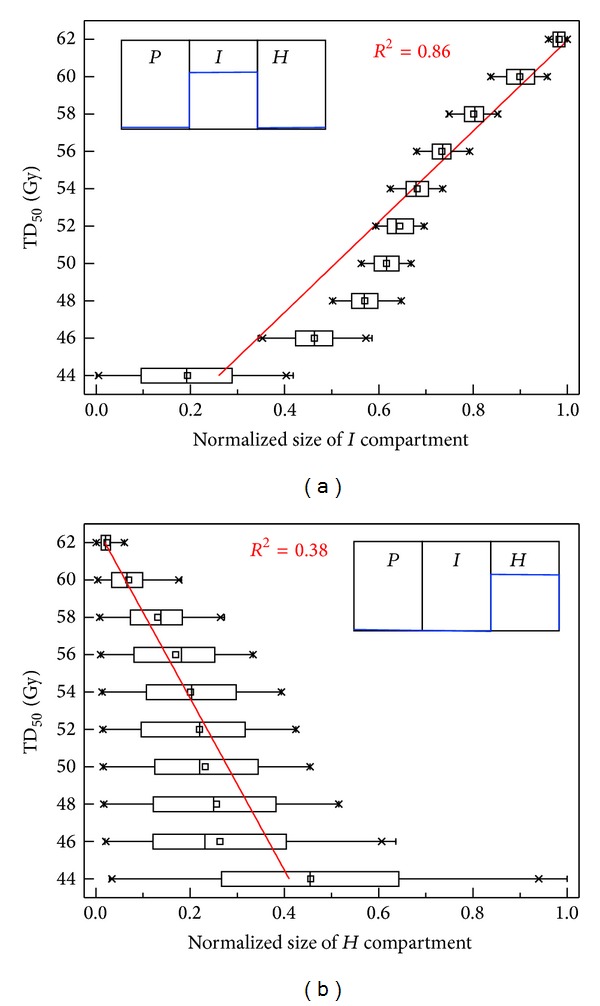
Model predicted tumor dose for 50% control (TD_50_) in 2 Gy/fx versus the number of cells in (a)* I*-compartment or (b)* H*-compartment. Note that each box in the plot represents 25th to 75th percentile of the dataset with tails for whole range.

We tested for any potential role of starving and dying cells (in the* H*-compartment). When the total number of cells in each hypoxic compartment (*I*- or* H*-compartment) was correlated with the required TD_50_ values, a strong positive correlation was observed for the* I*-compartment (*R* ^2^ = 0.86) but a weak negative correlation for the* H*-compartment (*R* ^2^ = 0.38), as shown in Figure 6.

This implies that the extremely hypoxic cells in the* H*-compartment are not the cause of an increased radioresistance. Mathematically, this is due to the lower OER value of the* H*-compartment used for the model simulation (OER_*H*_ = 1.37), compared to the OER of the* I*-compartment (OER_*I*_ = 2). To better understand this point, higher values of OER_*H*_ (OER_*H*_ = 2 or 3) were simulated (results not shown). Although the significance of the correlation was slightly reduced as the OER_*H*_ increased; quantitatively similar relationships were observed. Even when the OER_*H*_ was assumed to be as high as 3, which is certainly unreasonable radiobiologically, a strong correlation between the required TD_50_ and the intermediate hypoxia in the* I*-compartment was still present (*R* ^2^ = 0.83).

In this work, it was assumed that there is no proliferation in the* I*-compartment for simplicity. Although it is known that some cells can proliferate even in hypoxic condition [25], the effect was evaluated to be not that significant due to the trade-off between increased proliferation and increased mitotic cell death, as shown in the previous work [21].

The ratio of TD_50_ values between high versus low FDG uptake tumorlets, separated by median normalized FDG uptake, was estimated to be about 1.24 for the most likely uptake pattern,* pattern III*. This implies that high FDG uptake tumorlets require about 24% extra dose, which agrees with the clinically estimated range of the extra dose (~20%) for FDG-avid head and neck cancers [10]. However, this estimate might be an over-estimate of the effect, since it assumes maximum heterogeneity of CLF and GF parameters, as shown in Table 1.

The results suggest that the total number of metabolically viable hypoxic cells (in* I*-compartment of the model) is a deterministic factor in tumor response and this subpopulation might be associated with the voxel-by-voxel correlation studies performed by Pugachev et al. [16] and Rajendran et al. [15], in which the microregional relationship between FDG and hypoxia was observed. Also the result supports the study of Wouters and Brown, in which the importance of the cells at intermediate oxygen level was emphasized [26].

## 5. Conclusion

In this work, the potential relationship between FDG-PET uptake and classical radiobiological mechanisms was explored using a mathematical framework based on a law of conservation of chemical resources. Several different FDG uptake patterns were hypothesized and the estimated FDG uptake values were correlated with tumor dose for 50% control (TD_50_) using the state-driven tumor response model. The model could generate a correlation between FDG uptake and an increase in tumor radioresistance when it was assumed that cells receiving glucose but not oxygen take up more glucose than cells that are well-oxygenated. Establishing this correspondence further, and understanding its limitations, will require appropriate FDG-PET clinical datasets.

## Figures and Tables

**Table 1:** Parameter values for different compartments and the ranges of GF and CLF used in the model simulation.

Compartment	P	I	H
Oxygen enhancement ratio (OER)	1	2	1.37

Glucose uptake level per cell	Varies depending on the hypothetical FDG uptake pattern^a^		

Proliferation fraction	50–100%^b^	—	—

Cell loss mechanism and rate	Mitotic cell death due to irradiation (survival rate of each progeny after mitosis: 0.3)	—	Cell loss due to starvation (cell loss half time: 2 days)

Growth fraction (GF) range applied	0.01 to 1/(2 + CLF) [0.01 step]		

Cell loss factor (CLF) range applied	0.03 to 0.99 [0.03 step]		

^a^The relative uptake ratios in *P* : *I* : *H* are 1 : 1 : 0, 3 : 1 : 0, 1 : 3 : 0, and 2 : 5 : 2 for patterns I, II, III, and IV, respectively, as shown in Figure 2.

^b^Depends on the fullness of the compartment: 50% of proliferation was assumed when the compartment is full, and 100% of proliferation was assumed when the number of cells is less than half of the capacity of the *P*-compartment.
